# Comparison of TonoLab, TonoVet Plus, and Tono‐Pen AVIA Vet Tonometers for Measuring Intraocular Pressure in Rats

**DOI:** 10.1111/vop.70139

**Published:** 2026-01-14

**Authors:** Tarcísio Guerra Guimarães, Antônio Felipe P. F. Wouk, Karla Menezes Cardoso, Francisco Caramelo, Luís Carvalho, João Monteiro, Carlos Miguel Marto, António Francisco Ambrósio, Nuno Alexandre, Maria Filomena Botelho, Mafalda Laranjo

**Affiliations:** ^1^ Faculty of Medicine University of Coimbra, Coimbra Institute for Clinical and Biomedical Research (iCBR), area of Environment Genetics and Oncobiology (CIMAGO) and Institute of Biophysics, Faculty of Medicine Coimbra Portugal; ^2^ University of Évora, Institute for Advanced Studies and Research (IIFA) and Mediterranean Institute for Agriculture, Environment and Development (MED) Évora Portugal; ^3^ Center for Innovative Biomedicine and Biotechnology (CiBB) University of Coimbra Coimbra Portugal; ^4^ Division of Veterinary Ophthalmology, Department of Veterinary Medicine Federal University of Paraná (UFPR) and MeBi Veterinary Hospital Curitiba Paraná Brazil; ^5^ Clinical Academic Center of Coimbra (CACC) Coimbra Portugal; ^6^ Vet World Veterinary Clinic Figueira da Foz Portugal; ^7^ Faculty of Medicine and Centre for Mechanical Engineering, Materials and Processes (CEMMPRE), Advanced Production and Intelligent Systems (ARISE) University of Coimbra, Institute of Experimental Pathology Coimbra Portugal; ^8^ Association for Innovation and Biomedical Research on Light and Image (AIBILI) Coimbra Portugal; ^9^ Department of Veterinary Medicine University of Évora Évora Portugal

**Keywords:** applanation tonometry, rebound tonometry, tonometer, Wistar rat

## Abstract

**Objectives:**

To assess the agreement between intraocular pressure (IOP) measurements in rats using different animal‐adapted tonometers and to evaluate the relation among the devices.

**Methods:**

IOP was measured using the TonoLab tonometer, followed by the TonoVet Plus and finally, the Tono‐Pen AVIA Vet. A total of 24 eyes were studied from 12 healthy Wistar rats anesthetized with sevoflurane. Agreement analysis was performed between the TonoLab tonometer and the TonoVet Plus, and the TonoLab and Tono‐Pen AVIA Vet using the intraclass correlation coefficient (ICC). For the tested pairs, Bland–Altman graphs were also performed. The relation of TonoVet Plus and Tono‐Pen AVIA Vet tonometers to TonoLab was also evaluated using a simple linear regression.

**Results:**

The agreement between the TonoLab tonometers and the TonoVet Plus was weak (ICC = 0.173, 95% CI [−0.129; 0.489], *p* = 0.130) and not statistically significant. The agreement between the TonoLab and the Tono‐Pen AVIA Vet tonometers was negligible (ICC = 0.011, 95% CI [−0.117; 0.218], *p* = 0.448). Linear regression analysis between the TonoLab and the TonoVet Plus (*p* = 0.256), as well as between the TonoLab and the Tono‐Pen AVIA Vet (*p* = 0.897), was not statistically significant.

**Conclusions:**

The measurements revealed a consistent pattern in which the Tono‐Pen AVIA Vet showed the highest mean IOP values, followed by the TonoVet Plus and the TonoLab. Knowing the normal values of indirect IOP in rats for each of these tonometers, as well as the differences between them, is fundamental for the proper interpretation of the measurements obtained and for allowing reliable comparisons between the devices.

## 1 Introduction

The assessment of intraocular pressure (IOP) has been performed in various animal species [1, 2, 3, 4, 5] and in humans [6, 7]. Tonometry has been fundamental for the diagnosis and monitoring of ophthalmological diseases that may lead to irreversible vision loss. In this context, lower‐than‐normal IOP may be a sign of uveitis, while higher IOP may be a sign of glaucoma [8]. Thus, equipment capable of measuring IOP accurately and reproducibly has become essential to identify intraocular hypo‐ or hypertension, monitor disease progression, and evaluate treatment effectiveness [9, 10].

Measurement of IOP may be performed by manometry or tonometry [9, 11, 12]. However, all tonometric methods provide only indirect estimates, as the true IOP can only be obtained by direct cannulation of the anterior chamber of the eye, a procedure restricted to experimental settings [10, 13]. Consequently, clinical approaches rely on indirect estimation of IOP through tonometry [10].

The most common tonometers used clinically in animals are rebound tonometers and applanation tonometers, both of which display IOP in mmHg. Rebound tonometry determines the IOP via induction of an electromagnetic current generated by the rebound effect of a small probe onto the cornea [13, 14, 15], while applanation tonometry is based on the force required to flatten or applanate the central corneal surface, which is used to calculate IOP [13, 16], requiring topical anesthesia for IOP measurement.

Different portable tonometers have been designed for use in animals. The TonoLab, a rebound tonometer, has been designed for use in mice and rats. The TonoVet Plus (rebound) features specific calibration and modes for dogs, cats, horses, and rabbits. Both rebound tonometers must be placed perpendicular to the central area of the axial cornea. The TonoVet Plus has additional lights that indicate whether the probe is correctly aligned (green light) or not (red light). Tono‐Pen AVIA Vet, an applanation tonometer, has been designed to help eliminating the operator error and can function in any position, making it ideal for use in cats and dogs.

Although classified as exotic pets in veterinary practice, rats have proven to be valuable models for the pre‐clinical investigation of glaucoma [17, 18, 19]. As veterinary ophthalmologists often lack a specific tonometer for rat care, knowledge of normal values for each tonometer becomes important for proper result interpretation and accurate comparison of IOP outcomes obtained with different tonometers.

The present study has focused on comparing IOP values measured in Wistar rat eyes using different tonometers, namely TonoLab, TonoVet Plus, and Tono‐Pen AVIA Vet. The TonoLab tonometer was the only one designed and calibrated specifically for rats, with IOP values measured by the rebound tonometer showing good correlation with the manometric IOP [20, 21]. Despite the importance of establishing whether different commercially available equipments have agreement and calibration among them, no study to date has focused on comparative measurements outcomes. Accordingly, the present study aimed to evaluate the level of agreement in IOP measurements among the different devices.

## 2 Materials and Methods

### 2.1 Ethics Statement

The study herein was approved by the Animal Use Committee of the Faculty of Medicine, University of Coimbra (ORBEA 03/2018) and conducted in accordance with the Association for Research in Vision and Ophthalmology (ARVO), for the Use of Animals in Ophthalmic and Vision Research.

### 2.2 Animals Studied

Twelve Wistar female rats, 4‐month‐old, clinically and ophthalmically healthy, with a mean body weight of 224.7 g (SD ±17.3) and an axial globe length of 6.10 mm (±0.26), were included in the present study. Animals were maintained under controlled conditions of 32% humidity, 24°C, and a 12‐h light–dark cycle, with lights starting at 8:00 a.m. All rats were housed in transparent plastic rodent cages with ad libitum access to dry food and water at the Animal House of the Coimbra Institute for Clinical and Biomedical Research (iCBR), Faculty of Medicine, University of Coimbra.

### 2.3 Study Design

The same certified examiner performed all rat IOP measurements and handled different tonometers to avoid potential bias, with measurements taken between 11:30 a.m. and 3:30 p.m. Animals were anesthetized by placing them in an induction chamber with a 2.5% alveolar concentration of sevoflurane and 0.5 L/min oxygen. Immediately after sedation, rats were transferred to a via nose cone mask, and sevoflurane was supplied at the minimum necessary to keep the animal still and the eyeball centralized for IOP measurements. When eyeball rotation was present, anesthetic reduction was necessary until eye centring was achieved.

**FIGURE 1:**
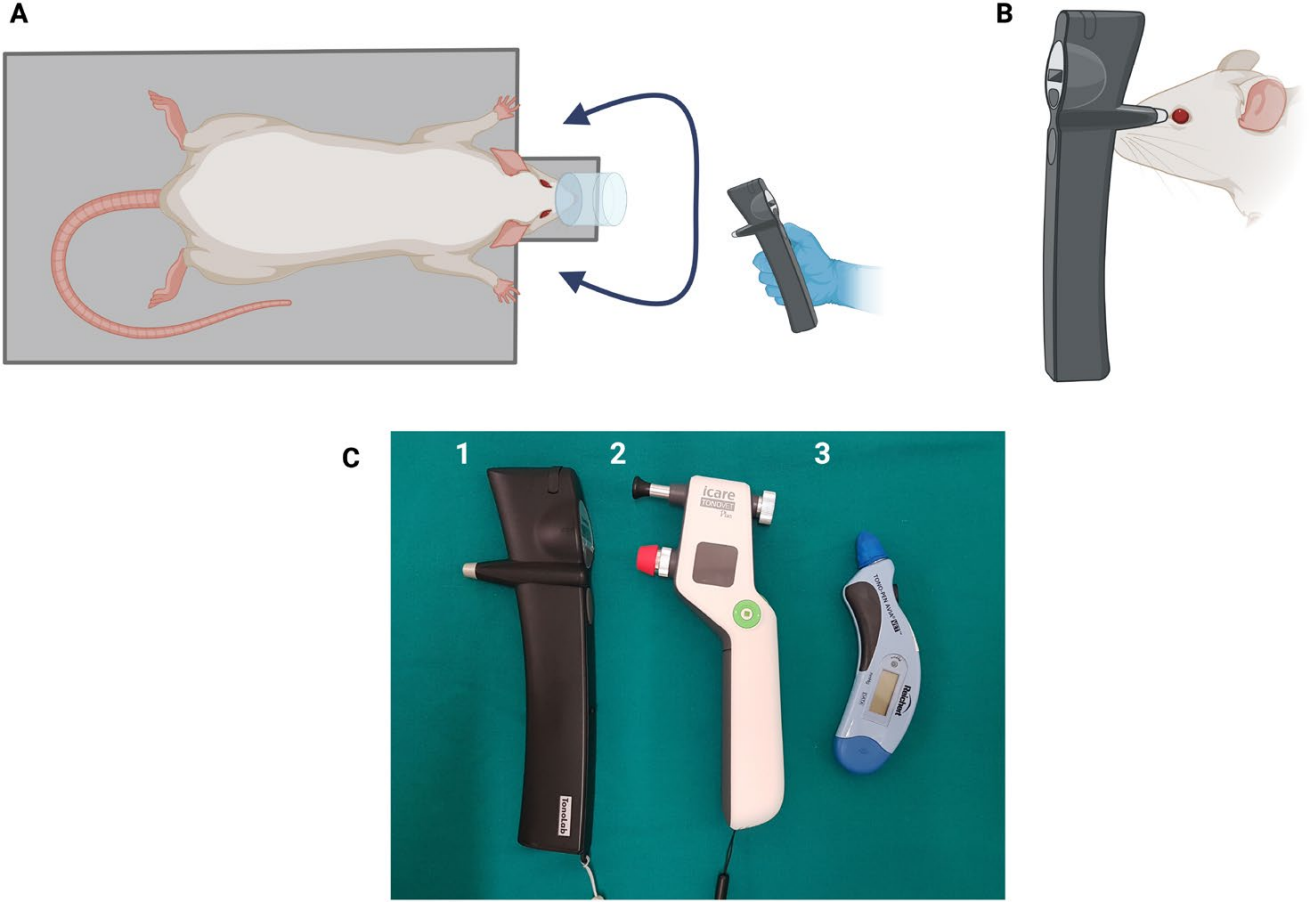
Study design. (A) Rat anesthetized and properly positioned in the platform. (B) Side view of the intraocular pressure measurement. (C) Tonometers assessed [(1) TonoLab, (2) TonoVet Plus, and (3) Tono‐Pen AVIA Vet]. Created in BioRender. Marto, C. (2026) https://BioRender.com/4q5465g.

The animals were positioned in sternal recumbency on a platform (Figure 1), allowing IOP measurement without interfering with the correct positioning and distance indicated for each tonometer.

**FIGURE 2:**
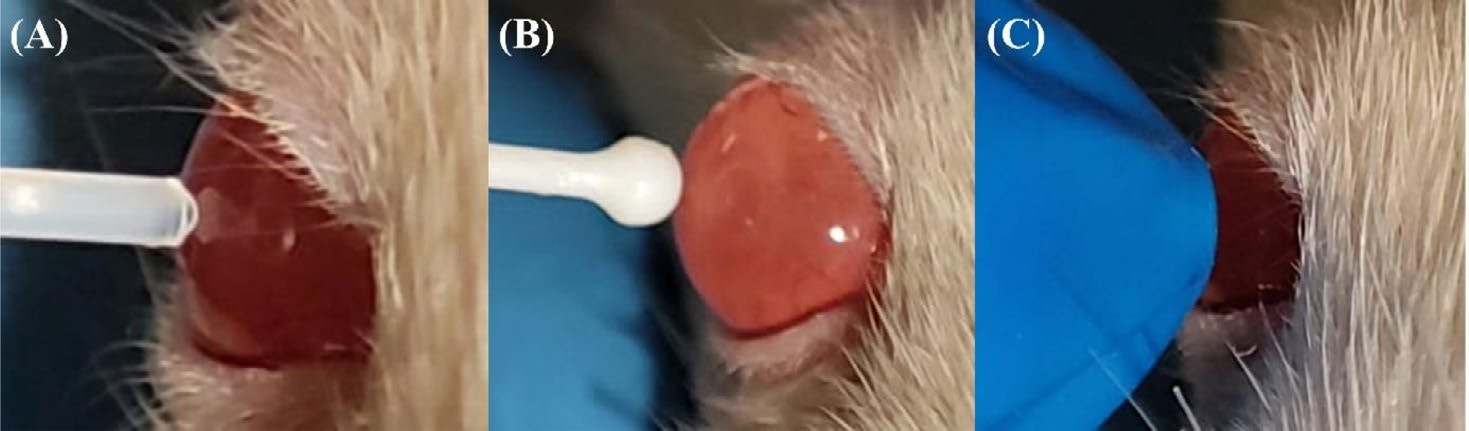
Measuring IOP in Wistar rat using different tonometers for use in animals, showing measurements in the axial region of the cornea. (A) TonoLab (rebound tonometer). (B) TonoVet Plus (rebound tonometer). (C) Tono‐Pen AVIA Vet with a blue Ocu‐Film tip‐cover (applanation tonometer).

All IOP measurements were obtained with identical animal handling, and all measurements were performed in the central region of the cornea (Figure 2). According to the standard protocol, examinations began with the right eye, followed by the left eye of each rat.

IOP was measured using the tonometer TonoLab (Icare TonoVet, Vantaa, Finland), followed by the TonoVet Plus (Icare TonoVet, Vantaa, Finland) and finally Tono‐Pen AVIA Vet (Reichert Inc., Depew, NY, USA). The measurement of each rat took less than 3 min, following the sequence of the three tonometers. The applanation tonometer was the last to be used because of the potential effect of topical anesthesia on rebound tonometry. Nonetheless, the mean IOP measured with a rebound tonometer has not been significantly altered in rats before and after topical anesthesia [22].

The TonoLab configured for rat measurement was brought close to their eyes, held in place by hand, and supported on the bench to minimize oscillations. The central groove of the TonoLab was in a horizontal position and at a distance of 1–4 mm from the tip of the probe to the central region of the cornea. Six measurements were consecutively taken and only considered when performed without any oscillations in the central part of the cornea and without any error message from the equipment.

The TonoVet Plus, configured for rabbit measurement, was brought close to the eyes of the animals in a horizontal position. The probe tip was positioned 4–8 mm away from the central region of the cornea, and the correct alignment of the probe was ensured with the aid of a green light indicator located at the base of the tonometer. Measurements presenting no error message or variations during the exam were considered, and the final IOP measurements were displayed on the screen, rounded by a green indicator (successful).

The Tono‐Pen AVIA Vet was applied using a latex membrane cover (Ocu‐Film). The Ocu‐Film was used according to the manufacturer's recommendations, keeping the rubber flat on the equipment tip and ensuring that the application was well‐fitted (neither too tight nor too loose). Before the IOP measurement, 10 μL of anesthetic eye drops (oxybuprocaine, 4 mg/mL) was applied to each eye with a micropipette. The tonometer was held perpendicular to the cornea so that the flat tip was parallel to the cornea. The central corneal surface was then discreetly touched without excessive pressure.

At the end of the IOP measurements, rats received a single lubricating eye drop (0.2% sodium hyaluronate) in each eye, and sevoflurane was discontinued. The rats then received oxygen flow until they had completely recovered from anesthesia.

### 2.4 Data Analysis

Agreement analyses were performed between the TonoLab tonometer, TonoVet Plus and Tono‐Pen AVIA Vet, using the intraclass correlation coefficient (ICC). Statistical power for ICC estimation was calculated using the ICC.Sample.Size package in R. The analysis examined the ICC values obtained. For these pairs, Bland–Altman plots were also performed. In addition, the relation between the TonoVet Plus and Tono‐Pen AVIA Vet with the TonoLab was also evaluated using simple linear regression. Statistical analysis was performed using IBM SPSS v27, with a maximum significance level of 0.05. The mean ± standard deviation (SD) was calculated for each tonometer, and a paired Student's *t*‐test was used to compare the IOP values between tonometers.

## 3 Results

Mean IOP ± SD and range for 12 Wistar rats (24 eyes) using TonoLab, TonoVet Plus, and Tono‐Pen AVIA Vet tonometers are shown in Table 1.

**TABLE 1:** Mean, SD and minimum and maximum intraocular IOP values in 24 eyes of 12 Wistar rats.

Tonometers	Mean IOP (mmHg)	±SD (mmHg)	Minimum IOP (mmHg)	Maximum IOP (mmHg)
TonoLab	13.58	±2.59	10	19
TonoVet Plus	15.63	±2.04	12	20
Tono‐Pen AVIA Vet	17.75	±2.11	13	22

No significant differences in IOP were found between the right and left eyes individually using either TonoLab, TonoVet Plus, or Tono‐Pen AVIA Vet (*p* = 0.438, *p* = 0.198, and *p* = 1.000, respectively).

**FIGURE 3:**
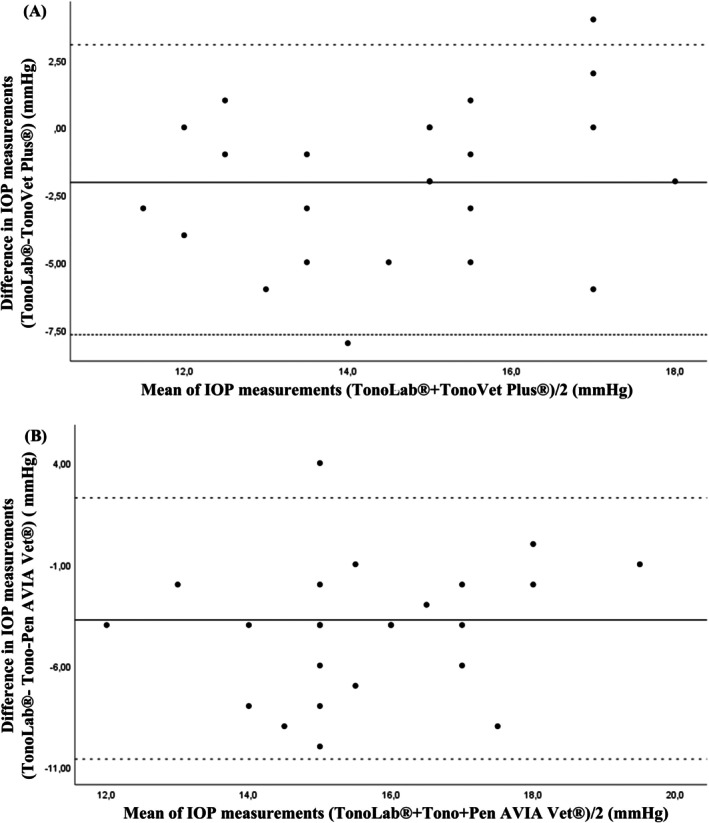
Bland–Altman plots between the TonoLab and TonoVet Plus tonometers (A) and between the TonoLab and Tono‐Pen AVIA Vet (B), in Wistar rats (*n* = 24 eyes). The mean difference was represented by the solid line (middle), and the upper and lower 95% limits of agreement were represented by the dashed lines on the top and bottom. Some data points are super‐imposed.

The agreement between the TonoLab tonometer and the TonoVet Plus was weak and not statistically significant (ICC = 0.173, *p* = 0.130), with an absolute agreement 95% CI of [−0.129; 0.489]. The Bland–Altman plot showed the discrepancies between the values from the two tonometers (Figure 3A).

**FIGURE 4:**
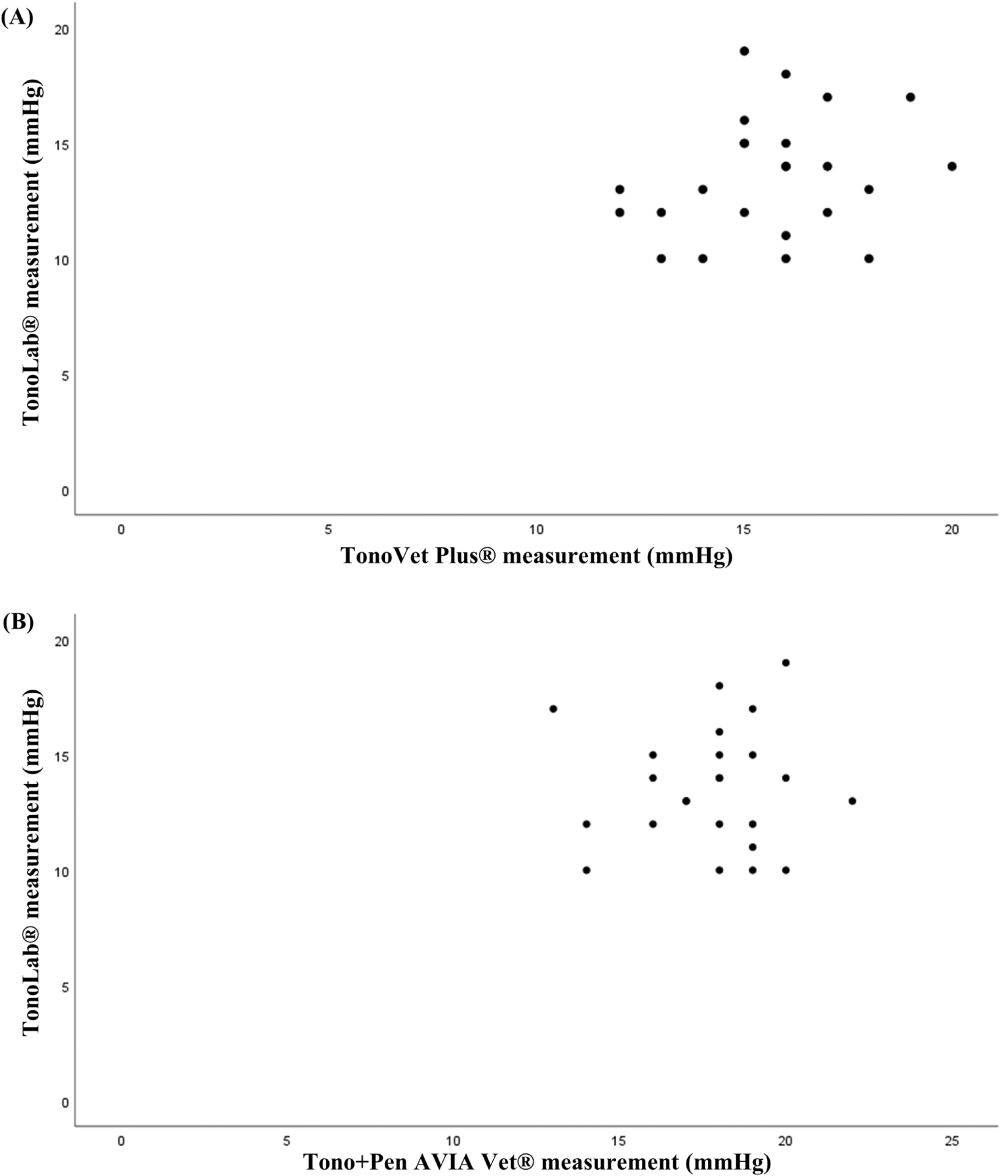
Scatterplot showing intraocular pressure readings and correlation obtained with TonoLab versus TonoVet Plus (A) and TonoLab versus Tono‐Pen AVIA Vet (B) tonometers. Some data points are super‐imposed.

The agreement between the TonoLab tonometer and the Tono‐Pen AVIA Vet was null (ICC = 0.011, 95% CI [−0.117; 0.218], *p* = 0.448). The Bland–Altman plot shows the discrepancies between the values obtained from the two tonometers (Figure 3B). Figure 4A shows the relation between the values of the TonoLab tonometers and the TonoVet Plus.

The regression between the values of the TonoLab tonometer and the TonoVet Plus was not statistically significant (*p* = 0.256), thus not allowing for the establishment of a calibration line between the values of the two tonometers. The graph between the values does not suggest the possibility of a non‐linear relation, preventing to obtain a calibration function between the two tonometers. Figure 4B shows the relation between the values of the TonoLab tonometer and the Tono‐Pen AVIA Vet.

The regression between the values of the TonoLab tonometer and the Tono‐Pen AVIA Vet was not statistically significant (*p* = 0.897), not allowing the establishment of a calibration line between the values of the two tonometers. The graph between the values does not suggest the possibility of a non‐linear relation, which precludes a calibration function between the two tonometers.

## 4 Discussion

As direct measurement of IOP is an invasive method, implying the penetration of the ocular bulbus [9, 23], non‐invasive indirect methods have been proposed to measure the IOP of animals [23]. In addition, rats and small rodents have become increasingly common as pets, and veterinary ophthalmologists often lack equipment specifically designed and calibrated for measuring IOP in these animals.

Although previous studies demonstrated and compared different tonometers in various animal species [24, 25, 26, 27, 28, 29], the study herein is the first to assess the agreement between tonometers exclusively for animal use, as well as the potential relation among them in rats.

Tonometry can usually be performed in most common domestic species, conscious animals, and in the clinical setting with minimal restraint [30]. In rodent that underwent successful behavioral training to accept conscious tonometry, measurement results were more consistent and reliable than in rodent under a restraint device [31]. Anesthesia can block the effects of stress on restricting rat movement, allowing for better estimates of IOP tonometry [32]. To minimize variation in our measurements due to stress or physical restraint, IOP was measured under volatile anesthesia with sevoflurane. Although no IOP measurements have been previously described in normal rats anesthetized with sevoflurane, sequential measurements with different durations of anesthesia with sevoflurane should be further investigated in rats, and the outcome may be useful in both clinical and experimental settings.

In adult humans, sevoflurane anesthesia does not significantly affect the values of the IOP measurements performed during wakefulness (without anesthesia), suggesting that sevoflurane is an adequate anesthetic for IOP assessment during required anesthesia [33]. Therefore, not surprisingly, IOP measurement in dogs anesthetized with sevoflurane presented no clinically significant differences compared to non‐anesthetized dogs [34]. In our study, rats underwent IOP measurements immediately after sedation, with a maximum duration of 3 min. Being that the most reliable measurement of IOP under anesthesia is obtained within the first minutes after induction, ideally within 3 min from the beginning of the procedure, before changes in IOP occur [35]. Although multiple IOP measurements have been recommended [36], multiple applications of the probe to the eye may iatrogenically affect IOP. Additionally, multiple measures are inconvenient and time‐consuming to perform [37, 38], consequently requiring more anesthesia time. The IOP should be checked as soon as possible after induction of anesthesia to obtain IOP measurements closest to the real IOP [39]. Still, IOP measurements are influenced by the circadian rhythm, as evidenced by peak values during the dark phase and trough values during the light phase [40, 41]. In the present research, all IOP measurements were performed during the light phase, respecting the schedules of the controlled laboratory environment.

Tonometers designed for animal use should be accurate across a wide range of species with different ocular anatomy [30], particularly in the veterinary clinical scenario, where different species of domestic, wild, and exotic animals may be submitted for ophthalmological evaluation. The accuracy of the applanation tonometry examination may depend on the corneal region and its biomechanical properties, as well as the force applied to the cornea [2, 30, 42, 43, 44, 45]. Thus, the rebound method may be subject to similar effects due to corneal properties [46]. Additionally, the absence or incorrect placement of the protection tip on the applanation tonometer can lead to inaccurate readings. In the present research, all rats were free of ophthalmological alterations, and measurements were performed with the indicated protective tip and adjusted according to the manufacturer's recommendations. Additional studies may include the analysis of corneal biomechanical properties to determine how central corneal thickness influences the measurements obtained by each tonometer. However, some studies indicate that there is no significant correlation between central corneal thickness and the recorded IOP values [2, 47, 48].

The difficulty of measuring IOP in small eyes, such as those of rodents, has been reported [49]. The rebound tonometers are easier to use on small eyes, such as those of rats. However, the position, alignment, and distance of the probe to the corneal tip may influence the IOP values at the time of measurement [50, 51]. The applanation tonometer made it possible to measure IOP in different positions [52]. To minimize potential sources of bias, including interexaminer variability, all IOP measurements were performed by the same experienced and trained examiner in the use of multiple tonometers in different animal species. This approach contributes to greater consistency and reliability of the data [50, 53].

Selecting the correct software, when available, has also been shown to be important for each species before performing tonometry [30]. Unspecific species selection during calibration can influence IOP results, since each configuration is based on specific assumptions about corneal biomechanics, potentially introducing systematic biases into the measurements [54]. However, the only commercially available equipment prepared for rat configuration was the TonoLab, the first tonometer available for exclusive rodent use since 2001 [55].

The mean IOP (±SD) obtained in Wistar rats was 13.58 mmHg (±2.59) using the TonoLab. Studies using the TonoLab in different species and strains of rats demonstrate that IOP values vary significantly depending on the strain, environmental conditions, and experimental protocols. Awake IOPs in 26 eyes of healthy F344 rats were 13.28 ± 2.56 mmHg, using the same equipment [49]. The measurements using the TonoLab tonometer in rats anesthetized with sevoflurane were similar to those in awake rats. Sprague–Dawley healthy and sedated rats, administered tiletamin and zolazepam, presented baseline IOP means (±SD) of 10.78 ± 1.43 mmHg, as measured using the TonoLab tonometer [22]. On the other hand, the resting IOP assessed by the TonoLab tonometer in conscious and physically restrained male Wistar rats was 18.4 ± 0.1 mmHg [21]. In awake Brown Norway rats housed in standard lighting, the TonoLab demonstrated significantly higher pressures during the dark phase (27.9 ± 1.7 mmHg) than during the light phase (16.7 ± 2.3 mmHg) [41]. In the present study, all measurements with the different tonometers were performed in the light phase.

The mean IOP (±SD) obtained in Wistar rats was 15.63 ± 2.04 mmHg using the TonoVet Plus. No reports of measurements with TonoVet Plus tonometry equipment were found in rats. However, a study measured the IOP in Guinea pigs ( *Cavia porcellus* ) using the TonoVet Plus reported mean ± SD values of 16.45 ± 1.34 mmHg for haired guinea pigs and 17.90 ± 2.04 mmHg for Skinny pigs using the TonoVet Plus [56].

The Tonovet, a previous version of TonoVet Plus, and configured for dogs, was applied in Wistar rats. The study evaluated the effect of different probe‐corneal distances during intraocular pressure data acquisition. Mean IOPs were 8.2, 9.4, and 10.5 mmHg using small (4 mm), medium (6 mm), and large spacers (8 mm), respectively [50]. The effect of the starting distance between the probe and cornea was not investigated in the present study, as a relatively constant distance was easily obtained. In addition, in all measurements with the different tonometers, the distance and form of use recommended by the manufacturer were respected.

Also, no previous work was found assessing IOP values in rats with the Tono‐Pen AVIA Vet applanation tonometer. In this study, the mean IOP (±SD) obtained was 17.75 ± 2.11 mmHg using the Tono‐Pen AVIA Vet. Similar values were obtained in IOP measured with Tono‐Pen (applanation tonometer human) in normal Lewis rats, with values of 17.30 ± 5.25 mmHg [57].

The average IOP values of the Tono‐Pen AVIA Vet were higher than those of the TonoVet Plus, which were higher than those of the TonoLab, in normal rat eyes.

Consistency between TonoLab and TonoVet Plus showed a weak intra‐class and not significant correlation (ICC = 0.173, *p* = 0.130), as both equipments measure IOP through rebound tonometry. When comparing the TonoLab with Tono‐Pen AVIA Vet, the agreement established was null (ICC = 0.011, *p* = 0.448). This result was expected due to the small size of the rats' eyes, in agreement with the findings of another study using an applanation tonometer [38], as Tono‐Pen AVIA Vet has the largest probe area in contact with the cornea (Figure 2).

The TonoLab and Tono‐Pen were calibrated in cannulated rat eyes connected to a pressure transducer. While TonoLab measurements were lower than those obtained with the transducer and Tono‐Pen across all IOP levels, they maintained an excellent linear correlation with transducer readings from 20 to 80 mmHg, with a correlation coefficient comparable to that observed between TonoLab and Tono‐Pen [41]. The TonoLab displayed greater accuracy and less variability than Tono‐Pen in measuring (manometrically determined) the IOP of 36 eyes of 18 female Wistar rats with a range of 9–20 mmHg [38]. In the present study, the highest values obtained with the TonoLab and Tono‐Pen AVIA Vet tonometers were 19 and 22 mmHg, respectively. Even at lower IOP values, the correlation between TonoLab and Tono‐Pen AVIA Vet was not achieved.

The TonoLab and TonoPen readings were strongly correlated with microneedle tonometer readings in Sprague‐Dawley rat eyes, but the TonoPen readings were only half those of the microneedle tonometer [58]. Nocturnal elevation of IOP was significant both with TonoLab and with the microneedle tonometer (*p* < 0.001), but not with TonoPen‐XL [58].

In normal male Wistar rat eyes, TonoPen overestimated manometric IOP at 10 mmHg and underestimated it by up to 6 mmHg at higher IOP, whereas the TonoLab matched set IOP within 1 mmHg [28]. A systematic review study analyzed the agreement between TonoLab, TonoVet, and TonoPen XL in Wistar rats. It was observed that TonoLab tends to underestimate IOP, while TonoPen XL tends to overestimate it compared to direct manometric measurement [59]. A weak agreement in the eyes of normal rats was observed between the TonoLab tonometers and the TonoVet Plus, and none between the TonoLab and the Tono‐Pen AVIA Vet. Further comparative studies involving different tonometers are warranted to establish their accuracy in small eyes, particularly in rats, laboratory animals and exotic pets, where ocular dimensions may significantly influence measurement reliability.

## Author Contributions

Conceptualization: T.G.G., A.F.P.F.W., M.F.B., and M.L.; data curation: T.G.G. and K.M.C.; IOP measurements: T.G.G.; anesthesia and anesthetic monitoring: K.M.C.; data record: L.C. and K.M.C.; figures performed: C.M.M., L.C., T.G.G., and F.C.; data analysis: F.C.; investigation: T.G.G., A.F.P.F.W., and K.M.C.; methodology: T.G.G., A.F.P.F.W., K.M.C., C.M.M., and M.L.; project administration: T.G.G., N.A., A.F.P.F.W., and M.L.; resources: M.F.B., A.F.A., J.M., and T.G.G.; supervision; T.G.G., A.F.P.F.W., N.A., M.F.B., A.F.A., and M.L.; validation: C.M.M., A.F.A., A.F.P.F.W., M.F.B., and M.L.; visualization: T.G.G., A.F.P.F.W., and C.M.M.; writing – original draft: T.G.G., C.M.M., A.F.P.F.W., and M.L.; writing – review and editing: C.M.M., F.C.S., N.A., A.F.A., A.F.P.F.W., M.F.B., and M.L. All authors have read and agreed to the published version of the manuscript.

## Disclosure

Artificial intelligence statement: The authors have not used AI to generate any part of the manuscript.

## Conflicts of Interest

The authors declare no conflicts of interest.

## Data Availability

The data that support the findings of this study are available from the corresponding author upon reasonable request.
